# Structural equation modeling of dietary patterns and association with vitamin D levels in children aged 9–16 years in Guangzhou, China

**DOI:** 10.3389/fnut.2024.1513376

**Published:** 2024-12-24

**Authors:** Jiaying Guo, Jie Huang, Shiyun Luo, Chunzi Zeng, Zheng Su, Jinhan Fu, Weiwei Zhang, Zhijun Bai, Zhoubin Zhang, Huilian Zhu, Yan Li

**Affiliations:** ^1^School of Public Health, Sun Yat-sen University, Guangzhou, China; ^2^Department of Foodborne Diseases and Food Safety Risk Surveillance, Guangzhou Center for Disease Control and Prevention, Guangzhou, China; ^3^School of Public Health, Southern Medical University, Guangzhou, China

**Keywords:** dietary patterns, vitamin D, children, China, SEM

## Abstract

**Background:**

Vitamin D deficiency and insufficiency represent critical public health concerns on a global scale. Due to the increase in indoor activities, the role of dietary intake of vitamin D has become increasingly prominent. However, previous studies have focused solely on a single food item.

**Objectives:**

This study aimed to identify dietary patterns among school-aged children in rural areas of Guangzhou, China, and to explore their association with vitamin D levels.

**Methods:**

A total of 2,346 children aged 6–17 years were included in this cross-sectional study. Demographic, lifestyle, and dietary data were collected through structured questionnaires. Dietary patterns were identified using factor analysis, while linear regression and structural equation modeling were employed to analyze the relationship between these patterns and vitamin D levels.

**Results:**

Three distinct dietary patterns emerged: a fruits and vegetables pattern, high-protein pattern, and snack pattern. Analysis revealed that a higher adherence to the fruits and vegetables dietary pattern was associated with lower vitamin D levels. Conversely, among girls, a stronger preference for the high-protein dietary pattern was positively correlated with higher vitamin D levels.

**Conclusion:**

The fruits and vegetables pattern emerged as a risk factor for inadequate internal vitamin D levels. In girls, the high-protein pattern functioned as a protective factor. These findings offer valuable insights and policy recommendations for enhancing the health status of children in rural communities.

## 1 Introduction

The high prevalence of vitamin D deficiency and insufficiency has been reported in numerous countries and is recognized as a major public health issue worldwide (1). Vitamin D deficiency has been linked to a range of acute and chronic conditions (2), such as rickets, osteomalacia, pre-eclampsia (3), impairments in immune function (4), and metabolic or cardiovascular diseases (5). Additionally, it is closely associated with children’s growth and development (6), infectious diseases, and autism spectrum disorders (7). This deficiency not only affects children’s immediate health but may also have long-term implications for the risk of future diseases in both children and adults, such as autoimmune diseases and cardiovascular diseases and obesity (8). It may even affect the development of children’s cardio-cerebral vascular systems, leading to abnormal psychological and behavioral development in children (9). The standard biomarker for assessing vitamin D nutritional status is the serum level of 25-hydroxyvitamin D (25(OH)D). The classification of vitamin D status—whether deficient, insufficient, sufficient, or excessive—is primarily based on the concentration of 25(OH)D. It is important to note that the optimal 25(OH)D level remains a subject of debate, with varying guidelines and organizations proposing different thresholds for defining vitamin D deficiency (10).

25(OH)D levels below 30, 50, and 75 nmol/L have been associated with median prevalence rates (95% CI) of 15.7% (13.7–17.8), 47.9% (44.9–50.9), and 76.6% (74.0–79.1), respectively, across global participants (11). A study involving 1,006 adolescents from 10 cities in nine geographically diverse European countries found that approximately 27% had 25(OH)D levels between 50 and 75 nmol/L, indicative of vitamin D insufficiency, while 15% exhibited levels below 50 nmol/L, signifying vitamin D deficiency (12). According to the “2015–2017 China National Nutrition and Health Status Monitoring Report,” the rate of serum vitamin D deficiency among children and adolescents aged 6–17 years in China was 18.6% during 2016–2017. A meta-analysis by Na et al. (13) revealed that the prevalence of vitamin D deficiency in Chinese children reached 24.03%, with an insufficiency rate of 28.71%, which increased with age. In rural areas, particularly those that are impoverished, the prevalence of vitamin D deficiency among school-age children significantly exceeds the national average (14). Thus, the current landscape of vitamin D deficiency and insufficiency, especially in rural regions, is critically concerning.

Various factors influence the body’s vitamin D levels, including reduced cutaneous synthesis, impaired absorption, and both acquired and genetic metabolic disorders (15). Dietary intake is a significant determinant of vitamin D status; in Denmark, 25(OH)D3 constitutes 24% of the dietary intake among children aged 4–17 years (16). A strong correlation exists between dietary vitamin D intake and vitamin D status in children (17). In Brazil, findings suggest that dietary protein intake may interact with genetic predispositions, affecting vitamin D levels (18). Jingrong et al. (19) discovered that daily consumption of fish and eggs, with an intake of ≥30 grams, can lower the risk of vitamin D insufficiency. Additionally, a 2-year prospective randomized trial demonstrated that a daily intake of 200 g of milk and 50 grams of eggs significantly improved growth and reduced vitamin D deficiency in children (20). The rise in indoor activities has led to decreased sunlight exposure for vitamin D synthesis (21), underscoring the growing importance of dietary intake for vitamin D nutritional levels. However, the majority of existing studies, which focus on the impact of single nutrients or foods on health, ignoring their complex interactions within the human body, which can influence bioavailability and absorption (22), providing relatively limited explanations for health outcomes.

Guangzhou, a prominent city in South China, is celebrated for its traditional Cantonese cuisine. However, research on the Lingnan dietary pattern and its health outcomes is relatively scarce, particularly among children. Additionally, in rural areas of Guangzhou, the issue of vitamin D deficiency in children is more pronounced due to factors such as economic conditions (23), the extent of nutritional knowledge dissemination, and lifestyle habits. Given that the proportion of urban infants and young children taking vitamin D supplements is significantly higher than that in rural areas, and considering that rural children may not meet the minimum dietary intake and dietary diversity (24), investigating the dietary patterns of children in rural areas is crucial for addressing the problem of vitamin D deficiency. Thus, this study aimed to establish dietary patterns encompassing various dimensions, including food types, nutrient composition, and their interactions. It can comprehensively consider the possible interactions between various foods and nutrients, and may detect the cumulative effects of multiple nutrients, which is more conducive to implementing nutritional interventions and has greater public health significance. The findings provide new insights into childhood micronutrient inadequacy and offers scientific evidence and policy recommendations to improve children’s health outcomes.

## 2 Materials and methods

### 2.1 Participants

**Figure 1:**
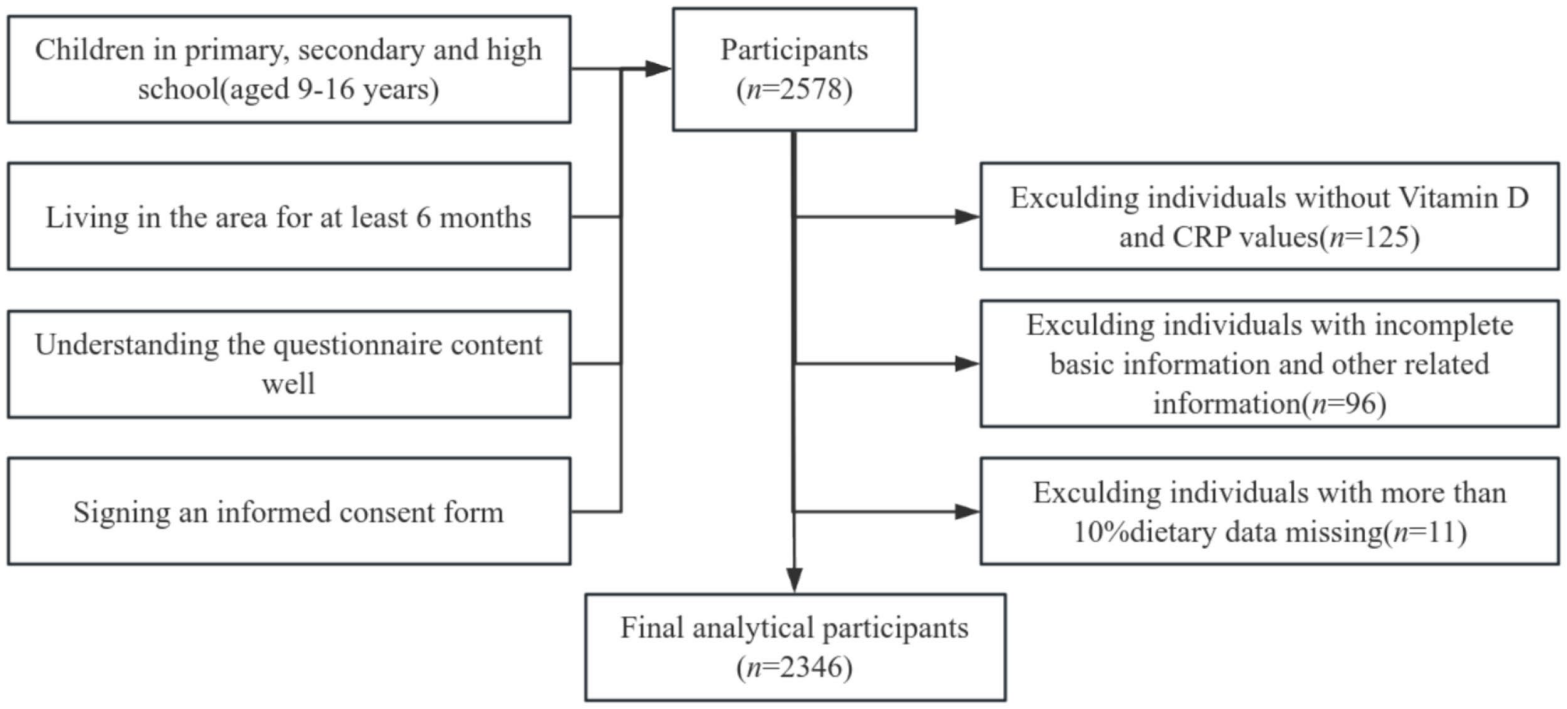
Selection process flowchart for research participants.

This study was conducted according to the guidelines laid down in the Declaration of Helsinki, and all procedures involving human subjects/patients were approved by the Ethics Committee of Guangzhou Center for Disease Control and Prevention (ethics number 2018027 and GZCDC-ECHR-2022P0038). All subjects and their guardians signed an informed consent form prior to being included in the survey. The cross-sectional study was conducted from May 2022 to June 2023. Participants were recruited using a multi-stage stratified cluster random sampling method: (1) five primary schools, five secondary schools, and two high schools were randomly selected from rural areas of Guangzhou. (2) Stratified sampling was employed, wherein 2–3 grades were randomly selected from each primary school, 2 grades from each secondary school, and 1 grade from each high school. (3) Additionally, 2–3 classes of students were randomly selected from each grade. The sample size was calculated using the following formula *N* = deff $\frac{u_{\frac{\alpha \;}{2}}^{2}P\;\left(1-P\right)}{\delta^{2}}$ (25). The meanings and values of each parameter are as follows: a confidence level of 95% (two-sided), $u_{\alpha /2}=1.96$ . The deficiency rate of vitamin D among Chinese children aged 6–17 years was noted to be 18.6% (26), with the probability P set at 19%. The design effect (deff) was set to 3, and the relative error (r) was determined to be 15%, yielding *δ* = 15% × 19%. Using these parameters, the initial calculation indicated a required sample size of 2,184 students. To account for invalid questionnaires and anticipated refusal rates, the sample size was increased by 10%, resulting in a final requirement of at least 2,402 students for participation. The selection process for survey participants is illustrated in Figure 1.

A total of 2,578 students were surveyed in this study. In alignment with the research objectives, cases with missing body measurement data and other relevant information were excluded, yielding a final sample of 2,346 students for analysis.

### 2.2 Survey content

In this survey, trained interviewers conducted face-to-face questionnaires with the subjects. Physical examination were centrally conducted on-site, and blood samples were sent to a unified laboratory testing institution for the analysis of serum vitamin D levels.

#### 2.2.1 Questionnaire survey

The questionnaire was segmented into four distinct sections: demographic details, lifestyle habits, nutritional knowledge evaluation, and dietary intake. It was grounded on the 2015 China National Chronic Non-communicable Disease and Nutrition Surveillance food frequency questionnaire (27), further refined through extensive expert consultations. This resulted in tailored adaptations to suit the local culinary preferences and dietary specifics of Guangzhou, integrating items that represent regional specialty foods. Throughout the dietary survey, precision in food weight estimation was enhanced by the use of food atlases and meticulous quality controls were implemented to ensure the integrity of data collection.

#### 2.2.2 Physical examination

The techniques for measuring height and weight adhered to the standards set forth in the “People’s Republic of China – Human Health Monitoring Anthropometric Methods (WS/T424-2013)” (28). These measurements primarily facilitated the calculation of body mass index (BMI). The assessment of nutritional status leveraged the 2007 World Health Organization’s age-specific BMI reference standards to ensure accurate categorization (29).

#### 2.2.3 Laboratory examination

The analysis of total serum vitamin D, encompassing D2 and D3 fractions, was conducted using the Watershed Acqui (liquid phase) liquid chromatography and Xevo-TQ-S mass spectrometry systems. This involved a liquid–liquid extraction method for analyte isolation, followed by ultra-high-performance liquid chromatography to mitigate serum matrix interference. Detection and quantification of these analytes, alongside their isotopic standards, were performed based on mass-to-charge ratios (m/z) using mass spectrometry. The isotopic internal standard method provided precise quantification of lipid-soluble vitamin D concentrations. Calibration was meticulously carried out using analytical standards from BePure, Achemtek, and Alfa, employing high-purity reagents like acetonitrile and methanol from Merck, n-hexane, and 2,6-di-tert-butyl-4-methylphenol. The coefficient of variance of intra- and inter assay were both <10%. The linear ranges were 0.78.0–78.41 ng/mL for 25(OH)D2 and 1.20–79.57 ng/mL for 25(OH)D3.

#### 2.2.4 Vitamin D definitions

Vitamin D status is categorized as deficient for serum 25(OH) vitamin D levels below 30 nmol/L, insufficient for levels between 30 nmol/L and 50 nmol/L, and sufficient for levels exceeding 50 nmol/L (30).

### 2.3 Establishment of dietary patterns

The dietary patterns were determined through exploratory factor analysis, which classified 126 food items into 15 predefined categories (Table 1). A comprehensive description of this analysis is available in prior literature (31). In this study, the Kaiser–Meyer–Olkin (KMO) test yielded a value greater than 0.87, while Bartlett’s test of sphericity produced a statistically significant result (*p* < 0.001).

**Table 1:** Food groups used in factor analysis.

Number	Food Group	Examples of food items
1	Cereal and tuber crops	Rice and its products, wheat and its products, corn and its products, potato and its products
2	Beans and its products	Soybean, soybean milk, tofu, bean curd, dried bean curd
3	Fresh vegetables	Cabbage, tomato, lettuce
4	Mushrooms and algae	Mushroom, laver, kelp
5	Fresh fruits and their products	Bananas, apples, berries, dried fruits
6	Milk and dairy products	Milk, milk powder, yogurt, cheese
7	Red meat	Pork, beef, goat, lamb, ham sausage, bacon, animal offal
8	Poultry	Chicken, duck, goose
9	Aquatic products	Fish, shrimp, crab
10	Eggs and its product	Eggs
11	Nuts	Peanuts, almonds, walnuts, hazelnuts
12	Candy	Sugar, jam, jelly, candies, chocolate, candied fruit
13	Convenience food	Instant noodles, instant rice noodles, cookies, cakes, bread, spicy strips, fried puffed snacks.
14	Fast food	Hamburger, fried chicken
15	Beverages	Carbonated drinks, prepackaged juice, milk beverages, sweet tea beverages, vegetable protein drinks, sports beverages, ice cream

### 2.4 Structural equation model

Structural equation modeling (SEM) was employed to establish, estimate, and test causal relationships among variables (32). This method consists of two components: the measurement model and the latent variable model. In this study, dietary patterns are treated as latent variables, whereas the intake levels of various food categories, age, sex, BMI, and vitamin D levels serve as observed variables. Vitamin D levels are represented by logarithmically transformed data, denoted as LnVD. Initially, confirmatory factor analysis was conducted to validate the measurement model of dietary patterns. Following this, the structural model examined the relationship between dietary patterns and LnVD. The parameter estimation method employed was maximum likelihood robust, with age, sex, and BMI included as adjusting variables. Model fit was evaluated using the chi-square to degrees of freedom ratio (CMIN/DF), comparative fit index (CFI), Tucker-Lewis Index (TLI), and root mean square error of approximation (RMSEA).

### 2.5 Statistical analysis

Details regarding the processing, coding, and validation of the questionnaire can be found in the referenced literature (33). Categorical variables are presented as *n* (%), while continuous variables, after undergoing normality testing, are described using the median and interquartile range (IQR). Linear regression analyses were conducted to explore the relationship between dietary patterns and logarithmically transformed vitamin D levels. Three models were fitted for each dietary pattern: an unadjusted model and two models adjusted for covariates. Model 1 represents the unadjusted analysis, Model 2 incorporates age and sex, and Model 3 includes age, sex, and BMI. The structural equation model was employed to investigate the correlation and degree of association between dietary patterns derived from factor analysis and logarithmically transformed vitamin D levels. Statistical analyses were performed using SPSS 26.0 and Mplus 8.3, with a significance level set at *p* < 0.05 (two-tailed). Data visualization was conducted using Microsoft Excel 2019.

## 3 Results

### 3.1 Participant characteristics

The study involved 2,346 participants, including 1,267 males (54.00%) and 1,079 females (46.00%). Participants’ ages ranged from 9 to 16 years, with a median age of 13.27 years (IQR: 11.31–14.38 years; Table 2). The prevalence rates of vitamin D deficiency and insufficiency were 13.43 and 46.63%, respectively. Most participants had parents with a high school education or lower. The distribution of boarding and non-boarding students was approximately equal. Regarding physical activity, 62.70% of participants were categorized as engaging in moderate to high levels of activity fewer than three times per week. Notably, most of the subjects had never experimented with smoking or alcohol consumption. Among the students, 60.06% reported daily screen time of less than 2 h. Alarmingly, nearly half of the students experienced insufficient sleep. The assessment of nutritional status revealed a coexistence of malnutrition and overnutrition, with rates of malnutrition, overweight, and obesity at 11.94, 10.66, and 6.94%, respectively.

**Table 2:** Demographic and lifestyle characteristics of the study participants.

Variable	Total
Age, median (IQR)	13.27(11.31, 14.38)
Sex, *n* (%)	
Male	1,267(54.00)
Female	1,079(46.00)
Education of father, *n* (%)	
High school or below	1,726(73.57)
Junior college or above	560(23.87)
Unknown	60(2.56)
Education of mother, *n* (%)	
High school or below	1,718(73.23)
Junior college or above	574(24.47)
Unknown	54(2.30)
Boarding, *n* (%)	
Yes	1,148(48.93)
No	1,198(51.07)
Moderate-to-high-intensity exercise, *n* (%)	
<3 times/week	1,471(62.70)
≥3 times/week	875(37.30)
Tried smoking, *n* (%)	
Yes	138(5.88)
No	2,208(94.12)
Try alcohol consumption, *n* (%)	
Yes	344(14.66)
No	2,002(85.34)
Screen time, *n* (%)	
≤2 h/day	1,409(60.06)
>2 h/day	937(39.94)
Sleep duration, *n* (%)	
Sufficiency	1,212(51.66)
Insufficiency	1,134(48.34)
BMI, *n* (%)	
Underweight	280(11.94)
Normal weight	1,653(70.46)
Overweight	250(10.66)
Obesity	163(6.94)

### 3.2 Dietary patterns

**Figure 2:**
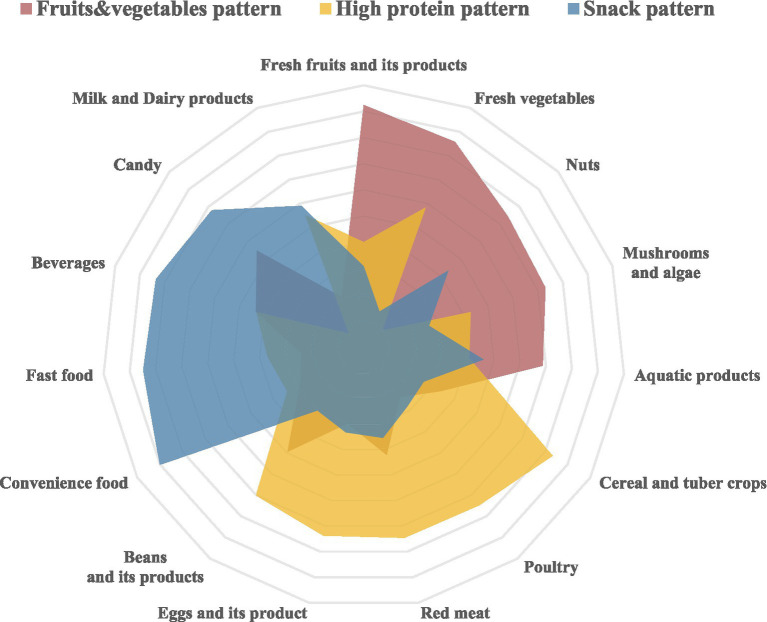
Radar chart depicting the various dietary patterns derived from factor analysis.

Factor analysis revealed three primary dietary patterns from the 15 food groups (Figure 2), accounting for 14.14% (fruits and vegetables pattern), 13.83% (high-protein pattern), and 13.41% (snack pattern) of the variance, collectively explaining 41.38%. Following rotation using the maximum variance method, the factor loading matrix for the food groups was established (Table 3). The fruits and vegetables pattern was characterized by fresh fruits and vegetables, supplemented by some nuts, aquatic products, mushrooms, and algae. The high-protein pattern, although primarily dominated by cereals and tubers, also included other protein-rich foods such as poultry, red meat, eggs, and legumes. The snack pattern was characterized by convenience foods, fast foods, beverages, and sweets, predominantly consisting of snacks that are high in energy but low in nutritional value.

**Table 3:** Factor loadings and dietary patterns for 15 types of food.

Food	Fruits and vegetables pattern	High protein pattern	Snack pattern
Fresh fruits and its products	0.724		
Fresh vegetables	0.657	0.384	
Nuts	0.542		
Mushrooms and algae	0.530		
Aquatic products	0.488		
Cereal and tuber crops		0.633	
Poultry		0.549	
Red meat		0.546	
Eggs and its product		0.538	
Beans and its products		0.501	
Convenience food			0.701
Fast food			0.648
Beverages			0.636
Candy	0.350		0.581
Milk and Dairy products		0.351	0.390

### 3.3 Association analysis between dietary patterns and vitamin D levels

#### 3.3.1 Analysis of dietary patterns and vitamin D levels

A linear relationship exists between the inclination levels of dietary patterns and logarithmically transformed vitamin D levels (Table 4). The findings indicate that a greater propensity toward the fruits and vegetables pattern is associated with lower logarithmically transformed vitamin D levels (*β* = −0.083, *p* < 0.001). Conversely, a stronger inclination toward the high-protein dietary pattern is associated with higher logarithmically transformed vitamin D levels (*β* = 0.088, *p* < 0.001). After univariate analysis, age, gender, and BMI were identified as covariates for adjustment. These results remain robust after adjusting for age, sex, and BMI. Specifically, the logarithmically transformed vitamin D levels show a significant negative correlation with the fruits and vegetables pattern (*β* = −0.103, *p* < 0.001) and a significant positive correlation with the high-protein pattern (*β* = 0.063, *p* < 0.05).

**Table 4:** Analysis of the association between dietary patterns and Vitamin D level.

Dietary Pattern	Model 1		Model 2		Model 3	
	*β*	95%CI	*β*	*95%CI*	*β*	95%CI
Fruits and vegetables pattern	−0.083***	(−0.019, −0.007)	−0.103***	(−0.022, −0.010)	−0.103***	(−0.022, −0.010)
High protein pattern	0.088***	(0.007, 0.020)	0.062*	(0.003, 0.016)	0.063*	(0.004, 0.016)
Snack pattern	0.028	(−0.002, 0.011)	0.003	(−0.001, 0.011)	0.032	(−0.001, 0.011)

*p < 0.05, **p < 0.01, ***p < 0.001. Model 1 remained unadjusted, Model 2 was adjusted for age and sex, and Model 3 was adjusted for age, sex, and BMI.

#### 3.3.2 Structural equation model

**Figure 3:**
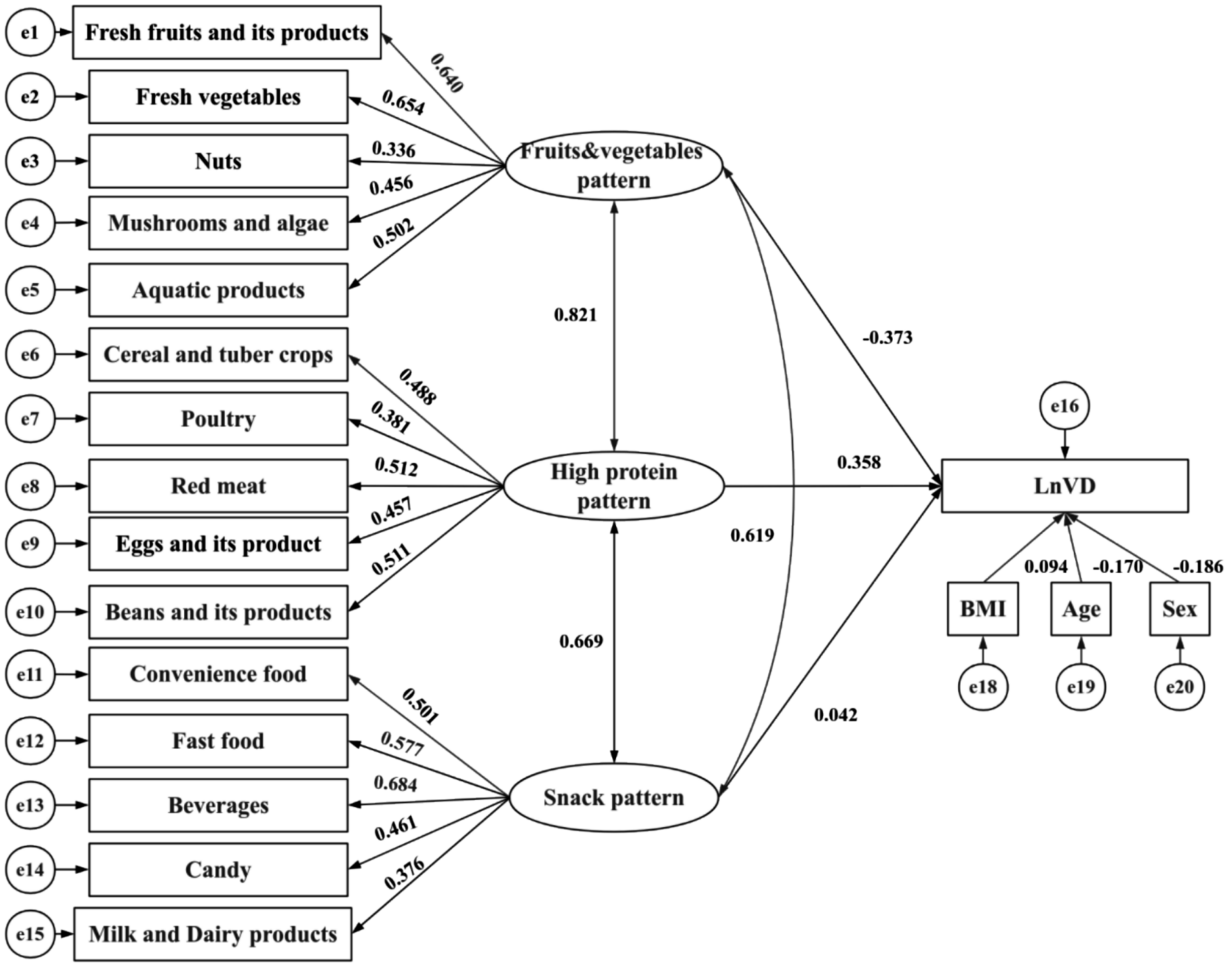
Structural equation models. The path standardized coefficients of variables are presented on pathways. CMIN/DF = 3.23, RMSEA = 0.033, CFI = 0.868, TLI = 0.847. Rectangles represent observed variables, while ellipses represent latent variables in the model.

The standardized estimates in the SEM diagram illustrate the relationships among the three dietary patterns and logarithmically transformed vitamin D levels (Figure 3). The goodness-of-fit indices for the model indicate a good fit (CMIN/DF = 3.23, RMSEA = 0.033, CFI = 0.868, TLI = 0.847), demonstrating a strong alignment between the theoretical model and the actual data regarding dietary patterns and logarithmically transformed vitamin D levels.

As shown in Figure 3 and Table 5, the fruits and vegetables pattern is significantly and inversely correlated with logarithmically transformed vitamin D levels (*β* = −0.373, *p* < 0.001). By contrast, the high-protein pattern exhibits a significant positive correlation with logarithmically transformed vitamin D levels (*β* = 0.358, *p* < 0.001). Covariates including sex, age, and BMI show statistically significant differences concerning logarithmically transformed vitamin D levels: age demonstrates an inverse correlation (*β* = −0.170, *p* < 0.001), while BMI is positively correlated (*β* = 0.094, *p* < 0.001). Notably, snack patterns do not exhibit any statistically significant association with the outcome. A pairwise correlation analysis reveals that the strongest correlation exists between the fruits and vegetables pattern and the high-protein pattern (*β* = 0.821, *p* < 0.001).

**Table 5:** Parameter estimates from the SEM of dietary patterns and Vitamin D.

Path analysis	Non-standardized coefficient	S.E.	C.R.	*p*-value	Standardized coefficient
Fruits and vegetables pattern → Vitamin D	0.000	0.000	−3.636	0.000	−0.373
Meat and egg pattern → Vitamin D	0.001	0.000	3.095	0.002	0.358
Snack pattern → Vitamin D	0.000	0.000	0.853	0.394	0.042

#### 3.3.3 Vitamin D levels in children of different genders based on dietary patterns

The results of the stratified analysis are presented in Tables 6, 7. Following stratification by gender, both boys and girls who displayed a greater inclination toward the fruits and vegetables dietary patterns were found to be more susceptible to lower vitamin D levels (*β* = −0.075, 95% *CI*: −0.018 to −0.003, *p* = 0.006 for boys; *β* = −0.141, 95% *CI*: −0.034 to −0.014, *p* < 0.001 for girls). Additionally, a positive correlation between high-protein dietary patterns and serum vitamin D levels was observed exclusively among girls (*β* = 0.088, 95% *CI*: 0.006 to −0.029, *p* = 0.003), with no corresponding association identified in boys.

**Table 6:** Association analysis for boys between dietary patterns and vitamin D levels.

Dietary Patterns	Model 1		Model 2		Model 3	
	*β*	95%CI	*β*	95%CI	*β*	95%CI
Fruits and vegetables pattern	−0.006	(−0.013, 0.002)	−0.076**	(−0.018, −0.003)	−0.075**	(−0.018, −0.003)
High protein pattern	−0.001	(−0.009, 0.006)	0.051	(0.000, 0.014)	0.054	(0.000, 0.015)
Snack pattern	0.001	(−0.006, 0.008)	0.031	(−0.003, 0.011)	0.028	(−0.003, 0.011)

*p < 0.05, **p < 0.01, ***p < 0.001. Model 1 remained unadjusted, Model 2 was adjusted for age, and Model 3 was adjusted for age, and BMI.

**Table 7:** Association analysis for girls between dietary patterns and vitamin D levels.

Dietary Pattern	Model 1		Model 2		Model 3	
	*β*	95%CI	*β*	95%CI	*β*	95%CI
Fruits and vegetables pattern	−0.127***	(−0.031, −0.012)	−0.141***	(−0.034, −0.014)	−0.141***	(−0.034, −0.014)
High protein pattern	0.090**	(0.006, 0.029)	0.088**	0.006, 0.029	0.088**	(0.006, 0.029)
Snack pattern	0.021	(−0.007, 0.015)	0.039	(−0.004, 0.018)	0.038	(−0.004, 0.018)

*p < 0.05, **p < 0.01, ***p < 0.001. Model 1 remained unadjusted, Model 2 was adjusted for age, and Model 3 was adjusted for age, and BMI.

## 4 Discussion

The present study showed the prevalence rates of vitamin D deficiency and insufficiency of 13.43 and 46.63%, respectively. The prevalence of vitamin D deficiency exhibits significant geographical variation, with particularly high rates reported in regions such as Asia, the Middle East, and Africa (34). In Asia, a widespread occurrence of infants with vitamin D levels below 30 nmol/L has been documented across several countries (34). For instance, in Turkey, the prevalence in this demographic reaches up to 51%, while in Iran, it escalates to 86%, underscoring the acute issue of vitamin D deficiency among infants and toddlers (34). Compared with other Asian countries, China’s situation is relatively better. A meta-analysis conducted by Na et al. (13) revealed that the prevalence of vitamin D deficiency in Chinese children is 24.03%, with an insufficiency rate of 28.71%, which shows an increasing trend with age. Compared with the results of Na et al.’s study, the findings of the present study indicate a significantly lower prevalence of vitamin D deficiency but a considerably higher prevalence of insufficiency than the national average. This suggests that, while the situation regarding vitamin D deficiency among school-age children has improved with ongoing socio-economic development (35), concerns remain significant.

This study employed dietary patterns to evaluate the nutritional status of children, offering a more holistic assessment that considers the complex interrelations among various dietary elements and nutrients (22). Three distinct dietary patterns were identified: a fruits and vegetables pattern, high-protein pattern, and snack pattern. These patterns provide a comprehensive framework for analyzing the nutritional intake of school-age children. The cumulative contribution rates of these three patterns are relatively balanced. Notably, as economic conditions improve, dietary patterns in China are gradually shifting from a traditional focus on fruits and vegetables to one characterized by increased protein intake, particularly from meat (36). Furthermore, the snack pattern, which primarily comprises fast food and convenience items, has emerged as a result of external cultural influences. The dietary patterns identified in this study can serve as critical nutritional guidance for children at risk of vitamin D deficiency.

In this study, multiple linear regression analysis indicated that the fruits and vegetables pattern was negatively correlated with logarithmic vitamin D levels, whereas the high-protein pattern exhibited a positive correlation. SEM corroborated these findings, demonstrating consistency between the two analytical methods and affirming the robustness of the results.

A comprehensive evaluation of the fruits and vegetables dietary pattern reveals several factors contributing to the observed lower levels of vitamin D, warranting careful consideration. First, despite including items such as certain seafood with relatively high vitamin D content, the overall intake remains low, as the primary constituents of this pattern are still predominantly fruits and vegetables. This reliance results in limited direct sources of vitamin D. Second, dietary fiber—particularly the insoluble variety abundant in fruits and vegetables—can increase intestinal content volume (37). This increase may lead to nutrient binding, including vitamin D, potentially reducing its solubility and absorption (38). Moreover, the relatively low fat intake associated with this dietary pattern could directly impede the solubility and absorption efficiency of vitamin D, as concurrent lipid co-transport is essential in the intestinal milieu. Lastly, the impact of dietary fiber extends beyond its direct effects; it is known to influence the metabolism and absorption of cholesterol and lipids (39), which, in turn, can indirectly affect vitamin D absorption by altering the lipid environment within the digestive tract. This indirect mechanism may further contribute to the reduced bioavailability of vitamin D. In summary, the dietary pattern’s reliance on fruits and vegetables, coupled with high dietary fiber and low fat intake, presents a multifaceted challenge for vitamin D absorption. This is in line with the findings of Neufingerl and Eilander (40), who observed that vitamin D intake associated with plant-based diets is typically inadequate, likely due to the limited number of foods naturally rich in vitamin D among fruits and vegetables (41).

By contrast, while cereals and tubers contribute significantly, the high protein dietary pattern is overwhelmingly dominant. A significant positive correlation exists between adherence to this pattern and vitamin D levels in the body, indicating that individuals who follow a high protein dietary regimen tend to have higher vitamin D levels. This finding can be reasonably attributed to the abundance of vitamin D3 sources within this dietary pattern. Specifically, it encompasses a variety of foods rich in vitamin D3, such as red meat, egg yolk, liver, and dairy products. Fish, particularly liver, contains the highest levels of vitamin D, followed by offal, which also provides substantial amounts of vitamin D (42, 43). Egg yolk ranks next, while red meat and meat products typically contain lower amounts. A common characteristic of these foods is their rich vitamin D3 content, alongside relatively high lipid levels. Given the fat-soluble nature of vitamin D, a high-fat environment enhances its dissolution and absorption in the intestine, thereby improving its bioavailability. The findings of this study align with existing literature, further reinforcing the positive correlation between this dietary pattern and vitamin D levels. For instance, a study in Taiwan has clearly indicated a link between adequate red meat consumption and a lower rate of vitamin D deficiency (44). This observation reflects the high protein pattern’s richness in red meat. Additionally, Polzonetti et al. (45) have highlighted the importance of whole milk, cheddar cheese, yogurt, butter, egg yolk, and certain mushrooms as significant dietary sources of vitamin D, all of which hold a prominent place in the high protein dietary pattern.

The gender-stratified analysis revealed a consistent decline in vitamin D levels among individuals adopting a fruits and vegetables dietary pattern, regardless of gender. This observation suggests that the high dietary fiber and phosphate content in fruits and vegetables may inhibit vitamin D absorption, indicating a potential universality in the mechanisms of action of these bioactive substances across genders. Further analysis showed that the high-protein dietary pattern exerts a protective effect solely in girls, likely due to the following reasons. Firstly, girls tend to engage in fewer outdoor activities compared to boys, which may reduce (46) their opportunities for sunlight exposure and subsequent vitamin D synthesis through ultraviolet radiation. Given the limited endogenous pathways for vitamin D synthesis, dietary components become particularly crucial for regulating vitamin D levels in girls. Secondly, studies have indicated that female intake of high-protein foods, particularly dairy products (47), is higher than that of males. This dietary preference suggests that girls may have a higher intake of vitamin D from food sources compared to boys, thereby potentially elevating their serum vitamin D levels. Thirdly, females generally have a higher body fat percentage than males (47). Given that vitamin D is a fat-soluble vitamin, the body composition of girls may facilitate better absorption and storage of vitamin D, enhancing the benefits of a high-protein diet that is rich in this nutrient (48). Lastly, the intricate role of sex hormones in vitamin D homeostasis can be considered. A negative correlation exists between estrogen levels and vitamin D status (49), while testosterone levels are positively associated with vitamin D concentrations (50). This hormone-dependent regulatory mechanism may amplify the influence of dietary patterns on vitamin D levels in girls, rendering the effects of nutritional interventions, such as high protein intake, more pronounced.

Hence, there is a need to place increased emphasis on health education for school-age children, offering scientific guidance in terms of dietary structure, quality, and habits. This approach can enhance the understanding of students, parents, and educational institutions regarding the importance of proper nutrition and balanced diets, modify unhealthy dietary behaviors, and encourage outdoor activities, all of which contribute to the healthy development of school-age children. In the case of school-age children from impoverished regions, taking into account the costs and benefits of interventions, the implementation of nutritional supplement programs may be considered as a feasible strategy to improve their nutritional status.

To the best of our knowledge, this study is the first to assess the effect of dietary patterns on vitamin D levels in school-age children, which is one of the most important indoor factors affecting vitamin D levels. The study has several strengths, the most important of which is its rigorous methodology and quality control. This study performed multi-stage cluster random sampling of subjects and multi-step quality control in the field data collection process, which enhanced the authenticity and validity of the results. Furthermore, multiple methods such as multiple linear regression, structural equation modeling, and stratified analysis were used to test the stability of the results. However, this study has three main limitations. First, the cross-sectional design of the research precludes the establishment of a clear causal relationship between dietary patterns and vitamin D levels. To validate the association, further longitudinal studies or intervention experiments are necessary. Second, data on lifestyle behaviors and dietary intake were collected through questionnaire surveys, a method that may introduce recall bias and social desirability bias. Finally, the study sample consists of rural children from the Guangzhou area, and the characteristics of this specific population may restrict the generalizability of the findings. When extending these results to other populations, it is crucial to account for the differences among diverse groups.

## 5 Conclusion

The relationship between dietary patterns and internal vitamin D levels merits further investigation. This study identified three distinct dietary patterns: the fruits and vegetables pattern, the high-protein pattern, and the snack pattern. Using dietary pattern factor scores for multiple linear regression and constructing structural equation models based on food intake amounts, validation of the results from both aspects makes the results more convincing. Notably, the fruits and vegetables pattern emerged as a risk factor for inadequate internal vitamin D levels. Conversely, among girls, the high-protein pattern functioned as a protective factor. The study findings provide robust scientific evidence and policy recommendations aimed at enhancing children’s health outcomes.

## Data Availability

The datasets presented in this article are not readily available because the data are not publicly available due to privacy. The data presented in the analyses for this study are available on request from the corresponding author. Requests to access the datasets should be directed to gzcdcliy@foxmail.com.

## References

[1] Amrein K Scherkl M Hoffmann M Neuwersch-Sommeregger S Köstenberger M Tmava A. Vitamin D deficiency 2.0: an update on the current status worldwide. Eur J Clin Nutr. (2020) 74:1498–513. doi: 10.1038/s41430-020-0558-y, PMID: 31959942 PMC7091696

[2] Bouillon R Manousaki D Rosen C Trajanoska K Rivadeneira F Richards JB. The health effects of vitamin D supplementation: evidence from human studies. Nat Rev Endocrinol. (2022) 18:96–110. doi: 10.1038/s41574-021-00593-z, PMID: 34815552 PMC8609267

[3] Jiayang X Guohui N. Research progress of vitamin D in neurodevelopmental disabilities. Med J Chin Peoples Lib Army. (2024) 49:586–593. doi: 10.11855/j.issn.0577-7402.0452.2024.0306

[4] Pereira M Dantas A Galvão L de T da J. Vitamin D deficiency aggravates COVID-19: systematic review and meta-analysis. Crit Rev Food Sci Nutr. (2022) 62:1308–16. doi: 10.1080/10408398.2020.1841090, PMID: 33146028

[5] Zhou A Selvanayagam J Hyppönen E. Non-linear Mendelian randomization analyses support a role for vitamin D deficiency in cardiovascular disease risk. Eur Heart J. (2022) 43:1731–9. doi: 10.1093/eurheartj/ehab809, PMID: 34891159

[6] Ganmaa D Bromage S Khudyakov P Erdenenbaatar S Delgererekh B Martineau AR. Influence of vitamin D supplementation on growth, body composition, and pubertal development among school-aged children in an area with a high prevalence of vitamin D deficiency. JAMA Pediatr. (2023) 177:32–41. doi: 10.1001/jamapediatrics.2022.4581 36441522 PMC9706398

[7] Shian Y Zhenyu Y. Status of vitamin a, vitamin D and comorbidity of both deficiency in Chinese children. Chin J Child Health Care. (2024) 32:301. doi: 10.11852/zgetbjzz2024-0146

[8] Xufei Z Zhibin J Yunzhi C. Epidemiological research progress on vitamin D deficiency in different populations in China. J Hubei Minzu Univ Ed. (2023) 40:75–9. doi: 10.13501/j.cnki.42-1590/r.2023.02.008

[9] Zittermann A Trummer C Theiler-Schwetz V Lerchbaum E März W Pilz S. Vitamin D and cardiovascular disease: an updated narrative review. Int J Mol Sci. (2021) 22:2896. doi: 10.3390/ijms22062896, PMID: 33809311 PMC7998446

[10] Demay MB Pittas AG Bikle DD Diab DL Kiely ME Lazaretti-Castro M. Vitamin D for the prevention of disease: an Endocrine Society clinical practice guideline. J Clin Endocrinol Metab. (2024) 109:1907–47. doi: 10.1210/clinem/dgae290, PMID: 38828931

[11] Cui A Zhang T Xiao P Fan Z Wang H Zhuang Y. Global and regional prevalence of vitamin D deficiency in population-based studies from 2000 to 2022: a pooled analysis of 7.9 million participants. Front Nutr. (2023) 10:1070808. doi: 10.3389/fnut.2023.1070808, PMID: 37006940 PMC10064807

[12] Mailhot G White JH. Vitamin D and immunity in infants and children. Nutrients. (2020) 12:1233. doi: 10.3390/nu12051233, PMID: 32349265 PMC7282029

[13] Na A Yile Z Guying Z Zhihua A. Meta analysis of vitamin D level in healthy Chinese children over the past 10 years. Chin J Child Health Care. (2021) 29:1109–14. doi: 10.11852/zgetbjzz2020-1798

[14] Yan S Hua G Bo H Linjuan L Shengnan H Wu. Status of vitamin D nutrition and its influencing factors among primary and middle school students in poverty areas of Guizhou, 2023. Mod Prev Med. (2024) 51:1466. doi: 10.20043/j.cnki.MPM.202402011

[15] Holick MF. Vitamin D Deficiency. N Engl J Med. (2007) 357:266–81. doi: 10.1056/NEJMra070553 17634462

[16] Jakobsen J Christensen T. Natural vitamin D in food: to what degree does 25-Hydroxyvitamin D contribute to the vitamin D activity in food? JBMR Plus. (2021) 5:e10453. doi: 10.1002/jbm4.10453, PMID: 33553993 PMC7839825

[17] Liu Y Li X Zhao A Zheng W Guo M Xue Y. High prevalence of insufficient vitamin D intake and serum 25-Hydroxyvitamin D in Chinese school-age children: a cross-sectional study. Nutrients. (2018) 10:822. doi: 10.3390/nu10070822, PMID: 29949856 PMC6073881

[18] Alathari BE Cruvinel NT da Silva NR Chandrabose M Lovegrove JA Horst MA. Impact of genetic risk score and dietary protein intake on vitamin D status in young adults from Brazil. Nutrients. (2022) 14:1015. doi: 10.3390/nu14051015, PMID: 35267990 PMC8912678

[19] Jingrong C Yinan Z Jie Z Yu T Qunying L Cheng L. Vitamin D status of children and adolescents aged 6-17 and its influence factors in some districts and counties, Chongqing. Mod Prev Med. (2021) 48:50–4.

[20] Zhao R Gan Q Hu Z Xu P Li L Yang T. Changes in fitness of rural primary school students from Southwest China after two-Year’s nutrition intervention. Nutrients. (2021) 13:3544. doi: 10.3390/nu13103544, PMID: 34684545 PMC8540577

[21] Sarathi V Dhananjaya M Karlekar M Lila A. Vitamin D deficiency or resistance and hypophosphatemia. Best Pract Res Clin Endocrinol Metab. (2024) 38:101876. doi: 10.1016/j.beem.2024.101876, PMID: 38365463

[22] Pk N Kl T. Empirically derived eating patterns using factor or cluster analysis: a review. Nutr Rev. (2004) 62:177–203. doi: 10.1301/nr.2004.may.177-203 15212319

[23] Zhigang C Jieying B Fengying N Xiangming F Shenggen F. New vision and policy recommendations for nutrition-oriented food security in China. Sci Agric Sin. (2019) 52:3097–107. doi: 10.3864/j.issn.0578-1752.2019.18.003

[24] Ke G Yuexin Y Tong L Yinhong Z Wei L Yu W. Improvement study of the vitamin a supplementary in school age children of 7 to 12 years old in rural area, Gansu Province. Chin J Dis Control Prev. (2018) 22:481–4. doi: 10.16462/j.cnki.zhjbkz.2018.05.012

[25] Zhan S Ye D Tan H. Epidemiology. People’s Health Publishing House (2017). 464 p. Available at: https://book.douban.com/subject/30218703/ (accessed October 30, 2023)

[26] Chinese Nutrition Society Health Management Branch. Expert consensus on evaluation and improvement of vitamin D nutritional status. Chin J Health Manage. (2023) 17:245–52. doi: 10.3760/cma.j.cn115624-20230105-00009

[27] Screening method for vitamin D deficiency in the population. Available at: https://ncncd.chinacdc.cn/gwxw/201506/t20150618_116195.htm (accessed August 19, 2024)

[28] Human body measurement methods for population health monitoring – National Health Commission of the People’s Republic of China. Available at: http://www.nhc.gov.cn/wjw/yingyang/201308/1f27caef0b22493e93a1da8aec2cd63a.shtml (accessed April 22, 2024)

[29] de Onis M Onyango AW Borghi E Siyam A Nishida C Siekmann J. Development of a WHO growth reference for school-aged children and adolescents. Bull World Health Organ. (2007) 85:660–7. doi: 10.2471/blt.07.043497, PMID: 18026621 PMC2636412

[30] Screening method for vitamin D deficiency in the population. (2020). Available at: https://std.samr.gov.cn/hb/search/stdHBDetailed?id=A701D7A974CFA3B7E05397BE0A0AEB89 (accessed July 28, 2024)

[31] Ma J Huang J Zeng C Zhong X Zhang W Zhang B. Dietary patterns and association with Anemia in children aged 9–16 years in Guangzhou, China: a cross-sectional study. Nutrients. (2023) 15:4133. doi: 10.3390/nu15194133, PMID: 37836416 PMC10574347

[32] Rb K. Latent variable path analysis in clinical research: a beginner’s tour guide. J Clin Psychol. (1991) 47:471–84. doi: 10.1002/1097-4679(199107)47:4<471::aid-jclp2270470402>3.0.co;2-o, PMID: 1939690

[33] Guo J Luo S Su Z Fu J Ma J Zhong X. Consumption patterns of sugar-sweetened beverages and association with undernutrition among children aged 9–17 years in Guangzhou, China: a cross-sectional study. Nutrients. (2024) 16:650. doi: 10.3390/nu16050650, PMID: 38474778 PMC10935377

[34] Roth DE Abrams SA Aloia J Bergeron G Bourassa MW Brown KH. Global prevalence and disease burden of vitamin D deficiency: a roadmap for action in low- and middle-income countries. Ann N Y Acad Sci. (2018) 1430:44–79. doi: 10.1111/nyas.13968, PMID: 30225965 PMC7309365

[35] Hao Z Yangyang D Ping M Wenqing D. Vitamin D nutritional status in Chinese children and adolescents: a meta-analysis. Chin J Evid-Based Med. (2021) 21:284–9. doi: 10.7507/1672-2531.202009106

[36] Su C Zhao J Wu Y Wang H Wang Z Wang Y. Temporal trends in dietary macronutrient intakes among adults in rural China from 1991 to 2011: findings from the CHNS. Nutrients. (2017) 9:227. doi: 10.3390/nu9030227, PMID: 28273878 PMC5372890

[37] Armet AM Deehan EC O’Sullivan AF Mota JF Field CJ Prado CM. Rethinking healthy eating in light of the gut microbiome. Cell Host Microbe. (2022) 30:764–85. doi: 10.1016/j.chom.2022.04.016, PMID: 35679823

[38] Weikert C Trefflich I Menzel J Obeid R Longree A Dierkes J. Vitamin and mineral status in a vegan diet. Dtsch Arzteblatt Int. (2020) 117:575–82. doi: 10.3238/arztebl.2020.0575, PMID: 33161940 PMC7779846

[39] Anderson JW Chen WJ. Plant fiber. Carbohydrate and lipid metabolism. Am J Clin Nutr. (1979) 32:346–63. doi: 10.1093/ajcn/32.2.346, PMID: 420130

[40] Neufingerl N Eilander A. Nutrient intake and status in adults consuming plant-based diets compared to meat-eaters: a systematic review. Nutrients. (2021) 14:29. doi: 10.3390/nu14010029, PMID: 35010904 PMC8746448

[41] Gaskins AJ Chavarro JE. Diet and fertility: a review. Am J Obstet Gynecol. (2018) 218:379–89. doi: 10.1016/j.ajog.2017.08.010, PMID: 28844822 PMC5826784

[42] Schmid A Walther B. Natural vitamin D content in animal products. Adv Nutr Bethesda Md. (2013) 4:453–62. doi: 10.3945/an.113.003780, PMID: 23858093 PMC3941824

[43] Dominguez LJ Farruggia M Veronese N Barbagallo M. Vitamin D sources, metabolism, and deficiency: available compounds and guidelines for its treatment. Meta. (2021) 11:255. doi: 10.3390/metabo11040255, PMID: 33924215 PMC8074587

[44] Huang Y-L Pham TTM Chen Y-C Chang J-S Chao JC-J Bai C-H. Effects of climate, sun exposure, and dietary intake on vitamin D concentrations in pregnant women: a population-based study. Nutrients. (2023) 15:1182. doi: 10.3390/nu15051182, PMID: 36904183 PMC10005797

[45] Polzonetti V Pucciarelli S Vincenzetti S Polidori P. Dietary intake of vitamin D from dairy products reduces the risk of osteoporosis. Nutrients. (2020) 12:1743. doi: 10.3390/nu12061743, PMID: 32532150 PMC7353177

[46] Yan X Zhang N Cheng S Wang Z Qin Y. Gender differences in vitamin D status in China. Med Sci Monit Int Med J Exp Clin Res. (2019) 25:7094–9. doi: 10.12659/MSM.916326, PMID: 31541605 PMC6767943

[47] Roshandel D Lu T Paterson AD Dash S. Beyond apples and pears: sex-specific genetics of body fat percentage. Front Endocrinol. (2023) 14:1274791. doi: 10.3389/fendo.2023.1274791, PMID: 37867527 PMC10585153

[48] Dong TS Luu K Lagishetty V Sedighian F Woo S-L Dreskin BW. A high protein calorie restriction diet alters the gut microbiome in obesity. Nutrients. (2020) 12:3221. doi: 10.3390/nu12103221, PMID: 33096810 PMC7590138

[49] Janssen HCJP Emmelot-Vonk MH Verhaar HJJ van der Schouw YT. Determinants of vitamin D status in healthy men and women aged 40-80 years. Maturitas. (2013) 74:79–83. doi: 10.1016/j.maturitas.2012.10.008, PMID: 23200514

[50] Chin K-Y Ima-Nirwana S Wan Ngah WZ. Vitamin D is significantly associated with total testosterone and sex hormone-binding globulin in Malaysian men. Aging Male Off J Int Soc Study Aging Male. (2015) 18:175–9. doi: 10.3109/13685538.2015.1034686, PMID: 26004987

